# Conductance estimation of a conductance-based neuron model by the differential evolution algorithm

**DOI:** 10.1186/1471-2202-14-S1-P212

**Published:** 2013-07-08

**Authors:** Mao Suzuka, Ryota Kobayashi, Katsunori Kitano

**Affiliations:** 1 Graduate School of Science and Engineering, Ritsumeikan University, Kusatsu, Shiga 5258577, Japan; 2 Department of Human and Computer Intelligence, Ritsumeikan University, Kusatsu, Shiga 5258577, Japan

## 

In the studies by a biologically detailed computational model of a neural circuit, it is an important step to construct the conductance-based model of a single neuron that reproduces electrophysiological data. Fitting model parameters such as the maximal conductances of ionic currents can be formulated as an optimization problem in which we should minimize the error between the data experimentally recorded and simulated by the model neuron. We here applied the differential evolution (DE) algorithm [1,2], one of evolutionary computation algorithms, to the parameter fitting problem based on the objective function defined by characteristics of an action potential profile.

We applied the DE algorithm to the conductance fitting to the synthetic data generated by a conductance-based model neuron. The model neuron consisted of spike-generating sodium, delayed rectifier potassium, A-type potassium and muscarinic potassium channels, conductance of which should be estimated. The objective function to be minimized was defined as a sum of six scores (distances to the target values) on action potential characteristics: firing rate, action potential peak, action potential width, depth of afterhyperpolarization, a latency to the first spike and degree of spike frequency adaptation [3]. We then compared its performance with that of the real-coded genetic algorithm (RCGA) as a benchmark under the same condition of population size and the number of generations.

**Figure 1:**
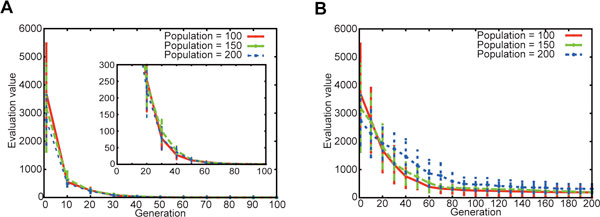
**Performances of conductance estimation by the DE and the RCGA**.

Figure 1 shows the time courses of values of the objective function, Evaluation values. Figure 1A illustrates averages and standard deviations over 10 trials at every 10 generations obtained by the DE with the population size of 100, 150 and 200. The inset of Figure 1A magnifies them in the lower range of the evaluation values. Figure 1B is similar to 1A, but by the RCGA. As indicated in the figures, the DE could minimize the evaluation value much more quickly than the RCGA. The DE reached the best value of 0.57 at 100^th ^generation and the value was achieved in all the trials. On the other hand, the best value by the RCGA was 29.29 only once out of the 10 trials. Thus, the results suggested that the DE had much better performance for the conductance fitting than the RCGA.
